# What the COVID-19 Pandemic Reveals about Racial Differences in Child Welfare and Child Well-Being: An Introduction to the Special Issue

**DOI:** 10.1007/s12552-021-09319-2

**Published:** 2021-02-08

**Authors:** Zachary Parolin

**Affiliations:** 1 Bocconi University, Milan, Italy; 2 Center on Poverty & Social Policy, Columbia University, New York, USA

**Keywords:** Race, Child well-being, COVID-19, Poverty, Families

## Abstract

This paper introduces the special issue on race, child welfare, and child well-being. In doing so, I summarize the evidence of racial/ethnic disparities in child well-being after the onset of the COVID-19 pandemic. Recent findings demonstrate that, compared to white children, black and Latino children are more likely to have experienced poverty and food insufficiency, to have had parents lose their jobs, and to be exposed to distance learning and school closures during the pandemic. I argue that though COVID-19 has indeed worsened racial/ethnic disparities in child well-being, it has also served to place a spotlight on the American welfare state’s historical mistreatment of low-income families and black and Latino families in particular. Consider that around three-fourths of black and Latino children facing food insufficiency during the pandemic also experienced food insufficiency prior to the onset of the pandemic. Moving forward, analyses of racial/ethnic disparities in child well-being during the pandemic, I argue, must not only consider the economic shock and high unemployment rates of 2020, but the failure of the American welfare state to adequately support jobless parents, and black and Latino parents in particular, long before the COVID-19 pandemic arrived.

In late 2019, when we began working on this Special Issue related to racial inequalities in child welfare and child well-being, we knew well that racial injustice was deeply entrenched into the public institutions of the United States. Black families, in particular, were more likely to encounter a child welfare system, criminal justice apparatus, education system, and a welfare state that each offered more hurdles and less support than for comparable white families. We had little idea at the time, however, of how the events of 2020 would only magnify and exacerbate those injustices. In spring of 2020, the COVID-19 pandemic prompted record levels of unemployment in the USA, hundreds of thousands of deaths, and widespread economic despair. And as with most crises in the USA, the socio-economic consequences of the pandemic did not hit evenly across race and ethnicity.

Consider that black and Latino children were much more likely than white children to experience poverty and/or economic hardship in the initial months after the pandemic (Couch et al. 2020; Gassman-Pines and Gennetian 2020; Hardy and Logan 2020; Parolin 2020; Vargas and Sanchez 2020). Black workers who lost their job are less likely than white workers to receive unemployment compensation, while non-citizens—a group that is heavily Latino in the USA—were barred from receiving stimulus checks during the crisis (Parolin et al. 2020b). Levels of food insufficiency among children—and black and Latino children in particular—are at alarming rates (Ziliak 2020). Asian Americans faced elevated rates of physical harassment and discrimination (Gover et al. 2020; Lee and Waters 2020). During the start of the new school year in autumn 2020, Asian, Latino, and black children were far more likely than white children to be exposed to school closures and distance learning, potentially exacerbating educational outcomes (Parolin and Lee 2020; Smith and Reeves 2020; Van Lancker and Parolin 2020). And each of these statistics, of course, only covers the living, but we must keep in mind that black and Latino individuals were more likely to die of COVID-induced symptoms in at least the initial months of the pandemic (Sze et al. 2020; Wrigley-Field 2020).

Put simply, the COVID-19 pandemic has brought racial/ethnic disparities in health, education, income, and general well-being into plain sight. But as scholars of racial differences in child well-being can attest, the racial/ethnic disparities capturing public attention today are far from new.

## The Great Revealer

One strand of economic historians argues that the economic shock imposed by a pandemic can contribute to reduced socio-economic inequality. In *The Great Leveler,* for example, Walter Scheidel details how the black Death of the 1300 s triggered a substantial decline in wealth inequality in Europe (Scheidel 2017).^1^ The COVID-19 pandemic, however, is hardly comparable in its effects. While it may eventually contribute to shifts in political preferences for more redistribution and a stronger welfare state, its short-run consequences, at least in the USA, have been to place a magnifying glass on the socio-economic challenges that black and Latino families, in particular, continue to face in the USA Rather than serving as *The Great Leveler,* the COVID-19 pandemic has, to this point, acted as *The Great Revealer.*

First, the pandemic has revealed to a broader audience that the American welfare state does not adequately support low-income families, nor does it treat black and Latino families the same as white families.

In the initial months after the pandemic, for example, media coverage and public attention were largely focused on the dysfunctions present within state-run unemployment insurance (UI) systems. The UI system is not simple to navigate: a mountain of administrative hurdles, particularly in states trying to reduce UI caseloads, contribute to a burdensome application process that many unemployed adults choose, at least in pre-pandemic times, to avoid altogether (Herd and Moynihan 2018). In many states, benefit payments to jobless adults are delayed by several weeks; many families are forced to scrape by in the meantime (Parolin et al. 2020b). Black and Hispanic workers, as noted, tend to be less likely to receive unemployment support, even in the midst of the pandemic (Edwards 2020; Hardy and Logan 2020).

Despite the increase in attention to the challenges of the UI system, however, the program’s dysfunctions are far from new. The primary difference now is that more Americans—including a new set of white workers with decent pre-pandemic incomes—have been forced to confront the UI system. As the less-marginalized join the ranks of their lower-income (often black and Latino) peers in navigating the American welfare state, calls for its overhaul have gained more traction. While such calls are welcome, families experiencing poverty and hardship prior to the onset of the pandemic would be right to question why, only now, their needs are deemed worthy of adequately addressing.

Consider the following. Before the onset of the pandemic, black and Latino children were around twice as likely as white children to live in poverty (Fox 2019). In fact, around 41 percent of black adults today spent at least half their childhood in poverty, compared to 5% for the average white adult (Corcoran and Adams 1997). Moreover, 17% of black and Latino families with children, on average, have experienced food insufficiency between April and November 2020, according to estimates from the Census Household Pulse Survey. But while framed in several studies as contributing to historic highs in hardship (Schanzenbach and Pitts 2020; Ziliak 2020), one should keep in mind that 72 percent of these same families report that they also experienced food insufficiency prior to the onset of the pandemic. Put simply, the American welfare state had been failing the vast majority of these black and Latino families long before the onset of the pandemic.

These racial/ethnic inequalities cannot be detached from the role of racism itself in influencing the comparative weakness of the American welfare state. A number of studies have documented the role of race and racism in reducing levels of income support for jobless adults (Alesina et al. 2001; Gilens 1999; Kohut et al. 2006; Michener 2018; Quadagno 1994; Schram et al. 2003; Soss et al. 2008). Even today, biases in state governments’ use of Temporary Assistance for Needy Families resources actively contributes to racial/ethnic differences in poverty: states with more black families, all else equal, are less likely to use their TANF resources for direct cash assistance, and more likely to spend the funds instead on influencing family formation (Parolin 2019). As a result, the black-white child poverty gap is larger than it would be if states’ were to use their TANF resources in a comparable way.

Meanwhile, non-citizen residents of the USA—a group that is heavily Latino—are barred from receiving many transfer benefits, including the stimulus checks offered after the onset of the pandemic (Parolin et al. 2020b). Put simply: the American welfare state has been tilted against black and Latino families for some time. It should be little surprise, then, that it is the same families facing the greatest levels of hardship after the onset of the pandemic. But there is one exception to this broader story of malaise: the four months in which the federal government provided the American welfare state with ample resources to invest in the nation’s residents.

This leads to the second point: the pandemic has revealed that the welfare state *can* work in reducing racial/ethnic differences in poverty and inequality—if the country adequately invests in it.

In late March 2020, the USA Congress passed the CARES Act, a massive spending package that provided direct cash payments to most lower-income (and many moderately high income) families, expanded access to unemployment benefits, and included a $600 per week unemployment supplement for those who could access unemployment benefits. The benefits were short-lived: the $600 supplements expired at the end of July 2020. Nonetheless, the Congressional Budget Office projected that the new income transfers would amount to more than $450 billion—more than the country spent on all direct income transfers combined in 2019 (Parolin et al. 2020b). Put simply, the CARES Act amounted to a massive increase in the size of the US welfare state.

What was the result? Over the four-month period in which the majority of benefits were distributed, poverty rates for all racial/ethnic groups actually declined (Parolin et al. 2020a). Specifically, in April 2020, a month in which unemployment spiked, the monthly poverty rate for black individuals fell 2.2 percentage points from its rate in January (23.8% to 21.6%), while the monthly poverty rate for Latino families fell 2.7 percentage points from January (23.7% to 21%). The overall child poverty rate fell from 18.7% in January to 15.5 percent in April. Again, the declines in poverty are directly attributable to the increase in income transfers.

The poverty reductions were short-lived: by August 2020, after the stimulus checks and unemployment supplement had expired, the monthly poverty rate increased to above 25% for black and Latino individuals—higher than rates observed before the onset of the crisis (Parolin et al. 2020a). The lesson, again, is rather clear: the American welfare state is capable of supporting its residents and contributing to declines in poverty, even during difficult times. But during the COVID-19 pandemic, the income support was pulled after only 4 months—even as the pandemic continued to worsen—leaving more than one in every four black and Latino individuals in poverty toward the end of 2020.

The pandemic has exacerbated racial/ethnic differences in child poverty and child well-being, yes. But more than anything, the pandemic has put a spotlight on the fact that the American welfare state insufficiently supported low-income families, and black and Latino families in particular, well before the pandemic arrived.

## A Preview of the Special Issue on Race, Child Welfare, and Child Well-Being

Put simply, racial injustice in the USA long predates the pandemic, and it will outlive it, as well. But the present moment offers yet more urgency to confront, with high-quality data and evidence, the systemic racial injustices that persist across America’s public institutions. Indeed, this is the purpose of this special issue. Several of the nation’s leading scholars on race, child welfare, and child well-being agreed to contribute to this issue of Race & Social Problems, and their combined contributions offer timely evidence not only of the disparities that currently exist across the American welfare state, child welfare system, and juvenile justice system, but also how such disparities can be confronted and reduced.

Three of the six papers in this Special Issue focus on racial inequality in the American welfare state. First, Dr. Carolyn Barnes and Dr. Lisa A. Gennetian investigate the experience of Hispanic families and social services in North Carolina. Using survey and qualitative data of administrators and front-line workers, the authors examine the unique challenges that Hispanic families face in accessing critical forms of income assistance that are known to reduce poverty and enhance child well-being. Their data demonstrate that national anti-immigration policies and rhetoric act as constraints on the capacity of service administrators to support Hispanic families.

Second, Dr. Diana Hernandez, Dr. George J. Musa, and a group of 16 other researchers present evidence on the role of housing subsidies in influencing adolescent mental health and well-being. Using data from 9–17 year olds from the Bronx, one of the poorest areas in the USA, the authors seek to understand how public housing and section 8 vouchers affect psychiatric symptoms and social functioning. They find that the lack of financial resources and time with family may contribute most to mental health disparities between children in subsidized versus non-subsidized housing.

Third, Dr. Megan Curran offers new findings related to the role of the US tax system in generating racial disparities through unequal provision of benefits from the Earned Income Tax Credit (EITC) and Child Tax Credit (CTC). Dr. Curran reveals that *family size* is an important yet understudied aspect of tax credit calculations that adds to the racial inequality in the tax system’s treatment of black and Hispanic families. Black and Latino children tend to live in larger families; meanwhile, the EITC and CTC benefit values are capped at three children and two children, respectively. The benefit caps, Dr. Curran finds, thus contribute to racial inequalities in child poverty.

The final three papers of the special issue turn attention toward the child welfare system.

Dr. Megan Feely and Dr. Emily Adlin Bosk investigate the need to bring a structural risk perspective to reduce racial disproportionality in child welfare. Specifically, the authors confront standardized decision-making procedures that aim to control for implicit or explicit bias in child protective systems (CPS). In doing so, they question how structural factors are accounted for in assessment of risk within CPS, and the consequences of leaving structural factors out of risk assessments procedures. The authors demonstrate that structural racism has been overlooked as an important cause of disproportionality in CPS and offer a revisioning of assessment of risk within child welfare that acknowledges the social determinants of CPS involvement.

Dr. Darcey Merritt uses data collected from qualitative research to highlight the lived experience of racism among families involved with the child welfare system. Her work directly incorporates the voices of black and Latino families, revealing how perceptions of race-based mistreatment generates feelings of disrespect, mistrust, and fear, particularly during the oversight and surveillance processes of the child welfare system.

The sixth paper in the special issue focuses on racial disparities in the juvenile justice system. Dr. Laura Abrams, Dr. Elizabeth S. Barnert, and Dr. Matthew Mizel apply a mixed-methods approach to examine rates of disproportionality among younger children in the California juvenile justice system. The authors find that at each stage of the juvenile justice system, the overrepresentation of black youth increases relative to white youth. The stakeholders they interview attribute this problem to early phases of the system (i.e., school discipline and arrests) where more discretion is applied. The authors conclude that minimum age laws that uniformly exclude younger children from the juvenile justice system altogether would mark a useful step toward reducing disparities in the juvenile justice system.

Together, these six studies confront systemic racial inequalities embedded into the American welfare state, child welfare system, and juvenile justice systems. The researchers offer findings that contribute to academic knowledge in their respective fields, but more importantly, they have offered concrete evidence and roadmaps toward reducing racial injustices in the USA I warmly invite policymakers, advocates, and fellow researchers who are concerned with racial inequalities in child welfare and child well-being to engage with, learn from, and act upon each of the papers included in this collection.

## Footnotes

1 For a counter-perspective on the historical role of pandemics in shaping inequality, see Alfani (2020).
